# Bilateral Retinal Angiomatous Proliferation in a Variant of Retinitis Pigmentosa

**DOI:** 10.1155/2019/8547962

**Published:** 2019-07-31

**Authors:** G. Aloe, C. M. De Sanctis, C. Strafella, R. Cascella, F. Missiroli, M. Cesareo, E. Giardina, F. Ricci

**Affiliations:** ^1^Unit Retinal Pathology PTV Foundation, Tor Vergata University, Rome, Italy; ^2^Department of Biomedicine and Prevention, Tor Vergata University, Rome, Italy; ^3^Molecular Genetics Laboratory UILDM, Santa Lucia Foundation, Rome, Italy; ^4^Department of Biomedical Sciences, Catholic University Our Lady of Good Counsel, Tirana, Albania

## Abstract

**Purpose:**

To describe the first case of bilateral retinal angiomatous proliferation (RAP) in a patient with a variant of retinitis pigmentosa (RP).

**Case Report:**

An 85-year-old man with RP presented with visual acuity decrease and metamorphopsia in the left eye (LE). Fundus examination revealed typical signs of RP in both eyes, associated with intraretinal macular hemorrhage in the LE. Multimodal imaging, using Colour fundus Photography, Fluorescein (FA), and Indocyanine Green Angiography (ICGA) as well as Spectral-Domain Optical Coherence Tomography (SD-OCT) and Optical Coherence Tomography Angiography (OCTA), revealed a type 3 neovascular lesion in the involved eye. Genetic testing (NGS analysis) was performed to search for genetic variants correlated with the disease phenotype displayed by the patient. The patient was treated with intravitreal injections of bevacizumab, according to a fixed protocol of bimonthly injections plus a booster dose at second month. After 9 months, he was referred for visual acuity decrease and metamorphopsia in the fellow eye, where SD-OCT/OCTA showed a type 3 neovascular lesion in the right eye (RE). He was scheduled for intravitreal injections of bevacizumab. In both eyes, treatment with intravitreal bevacizumab was successful.

## 1. Introduction

Retinitis pigmentosa (RP) is a complex of inherited retinal dystrophies characterized by the loss of photoreceptors and characterized by retinal pigment deposits in the peripheral retina with a relative sparing of the central retina. The most common form of RP is a rod-cone dystrophy, in which there is a primary degeneration of the photoreceptor rods, with secondary degeneration of cones, thus leading to night blindness, followed by the progressive loss in the peripheral visual field in daylight, and eventually blindness after several decades [1]. Retinal angiomatous proliferation (RAP), also referred to type 3 neovascularization, is a common neovascular lesion type occurring in age-related macular degeneration (AMD). It was first described by Yannuzzi et al in 2001 and it is characterized by intraretinal neovascularization with proliferation of intraretinal capillaries within the deep retinal layers, producing intraretinal and superficial retinal hemorrhages that can evolve toward a subretinal neovascularization and choroidal neovascularization (CNV) [2].

CNV is very rarely associated with RP [3]. To date, two cases of RP with RAP have been reported in literature, but those cases were not genetically characterized. Our study represents the first description of a bilateral type 3 neovascularization in a patient with RP genetically determined.

## 2. Case Report

An 85-year-old with RP was referred to our center in April 2016 for decrease in visual acuity and metamorphopsia in the left eye (LE) for a few weeks. The patient had the clinical diagnosis of retinitis pigmentosa from the age of 15 years old and for this reason did not do military service. He reported a history of night blindness and narrowing of the subjective visual field in both eyes with a recent visual impairment in the LE. Best corrected visual acuity was 0.1 LogMAR (80 LN) in the right eye (RE) and 0.7 LogMAR (50 LN) in the LE. The visual field test was not reliable due to the poor collaboration of the patient.

Fundus examination revealed arteriolar attenuation, peripapillary atrophy, optic disc pallor, macular edema and typical retinal pigment deposits involving the midperipheral retina (bone spicules) of both eyes, associated with intraretinal hemorrhage in the macular region of the LE.

Fluorescein angiography (FA) (Heidelberg Retina Angiograph II, Heidelberg Engineering, Heidelberg, Germany) revealed a continuous retinal-retinal anastomosis and the presence of well distinct area of intense leakage temporally to the fovea corresponding to the neovascularization with a late pooling of dye due to intraretinal cystoid edema [2]. In the fellow eye, FA showed late pooling due to cystoid macular edema.

Indocyanine green angiography (ICGA) (Heidelberg Retina Angiograph II, Heidelberg Engineering, Heidelberg, Germany) revealed a well-distinct “triangular shape” area of intense hypercyanescence (“hot spot”) corresponding to the neovascularization and a feeder and draining vessel, pathognomonic for RAP. In the fellow eye, ICGA did not show any signs of neovascularization.

Spectral-Domain Optical Coherence Tomography (SD-OCT) (Spectralis HRA OCT; Heidelberg Engineering, Heidelberg, Germany) in correspondence of the hyperfluorescent area of the LE revealed a hyperreflective intraretinal lesion located at the outer retinal layers apparently connected with the sub-RPE space, characterized by intraretinal and subretinal exudation and intraretinal fluid [4]. In the fellow eye, SD-OCT showed epiretinal membrane with cystoid intraretinal fluid.

Optical Coherence Tomography Angiography (OCTA) (Optovue, Inc., Freemont, CA, USA) is a rapid, noninvasive technique that showed clearly the intraretinal neovascularization with capillary proliferation and retinal-retinal anastomoses [5].

**Figure 1:**
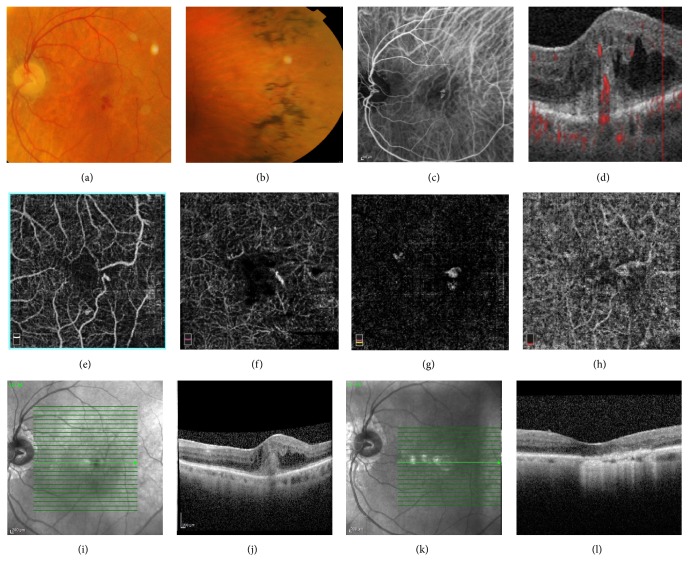
(a)-(b) Colour fundus photography showed arteriolar attenuation, peripapillary atrophy, optic disc pallor, macular hemorrhage and typical retinal pigment deposits (bone spicules) involving the mid-peripheral retina (c) Indocyanine green angiography (ICGA) revealed a well-distinct “triangular shape” area of intense hypercyanescence (“hot spot”) corresponding to the neovascularization (d) B-Scan optical coherence tomography angiography revealed high-flow intraretinal neovascularization originated in the superficial layer descending into a tuft-shaped lesion toward the sub-RPE space. (e)–(h) Optical Coherence Tomography Angiography images (6 × 6 mm) revealing the progression of the tuft-shaped lesion from the superficial layer to choroid capillary. (e) Superficial capillary plexus segmentation. (f) Deep capillary plexus segmentation. (g) Outer retinal layer segmentation. (h) Choriocapillaris segmentation. (i)–(j) Spectral Domain-Optical Coherence Tomography at baseline. (k)–(l) Spectral Domain-Optical Coherence Tomography after treatment.

In the segmentation of deep capillary plexus, OCTA revealed a high- flow third- order vessel descending into a tuft-shaped high-flow lesion toward the outer retinal layers, characterized by a retinal-retinal anastomosis and bordering the sub-RPE space [6] (Figure 1).

Taking into consideration the clinical picture of patient we decided to perform a genetic testing to search for genetic variants, which may be correlated with the RP-like phenotype observed in the patient. To this purpose, the patient was asked to sign an informed consent and to give a saliva sample for genetic testing [7]. The DNA was extracted using MagPurix Tissue DNA Extraction Kit and MagPurix Automatic Extraction System (Resnova) according to the manufacturer's instructions. Samples were sequenced by Next-Generation Sequencing (NGS) with Ampliseq DNA protocol and Ion Torrent S5 system. In this case, the panel contains 24 genes, which are associated with different anterior and posterior segment eye disorders, including Glaucoma, Retinitis Pigmentosa [8, 9].

NGS analysis revealed the presence of two missense variants (namely, NM_001193300: c.1265C>T and NM_001142564: c.1162T>C) located in SEMA4A (1q22) and in CNGA1 (4p12), respectively. In particular, SEMA4A encodes a member of semaphorin family of transmembrane proteins and modulate the transport of retinoid-binding proteins in RPE cells (REF4). CNGA1 codes for the homonymous protein that is crucial for phototransduction pathway [9]. The c.1265C>T variant (rs145228550) was found at the heterozygous state and resulted in an amino acid change (p.Thr422Ile). Multiple computational evidence predicted no impact on the gene or gene product, although it has a very low frequency (ExAc:0.00005) in the general population. The c.1162T>C variant (rs914018863) was detected at the homozygous state and caused an amino acid change (p.Tyr388His). Interestingly, c.1162T>C has a very low frequency (GnomAD <0.00001) and the prediction analysis reported a potential deleterious effect on the gene or gene product. Although c.1265C>T and c.1162T>C are located within two genes associated with retinal disorders including autosomal recessive RP, these variants are not described in literature and there are no functional assays demonstrating their real pathogenic effect. These variants are therefore classified as Variants of Uncertain Significance (VUS, class 3) according to the ACMG guidelines [10], meaning that such variants cannot be certainly classified as benign or pathogenic at the moment. Therefore, these results cannot be utilized to provide a genetic explanation to RP-associated phenotype observed in the patient, although the identified VUS may be revisited along with further investigations which may increase the knowledge concerning their clinical implications.

Successively, the patient was screened for AMD-related variants, in order to estimate the genetic susceptibility to disease, considering the observation of a neovascular lesion and the advanced age of the patient. In particular, three polymorphisms were tested, namely rs1061170 (T/C), rs10490924 (G/T) and rs2227306 (C/T) [11, 12]. They are located in CFH (1q31), ARMS2 (10q26) and IL8 (4q13) genes, respectively, which are known to be three major contributors to genetic susceptibility to AMD among Italian and worldwide populations [13, 14]. The test was performed by TaqMan assay using a 7500 Fast Real Time PCR device (Applied Biosystems) according to the manufacturer's instructions. Results were interpreted using Sequence Detection System 2.1 software (Applied Biosystems). The screening test revealed that the patient is heterozygous for the ARMS2 (G/T), IL-8 (C/T) and wild-type for CFH (T/T) polymorphisms, meaning that the patient is approximately 5 times more likely to develop AMD during his lifetime. These results highlighted therefore a genetic predisposition to develop AMD in the patient, which is consistent with the presence of a type 3 neovascularization.

We decided to treat the patient with 3 consecutive monthly intravitreal injections of bevacizumab adopting the Treat and Extend (TE) strategy after the loading phase.

In January 2017, 9 months later the first visit, patient referred for sudden visual acuity impairment in the fellow eye. Best corrected visual acuity decreased from 0.1 LogMAR 80 LN to 1.0 LogMAR 35 LN.

**Figure 2:**
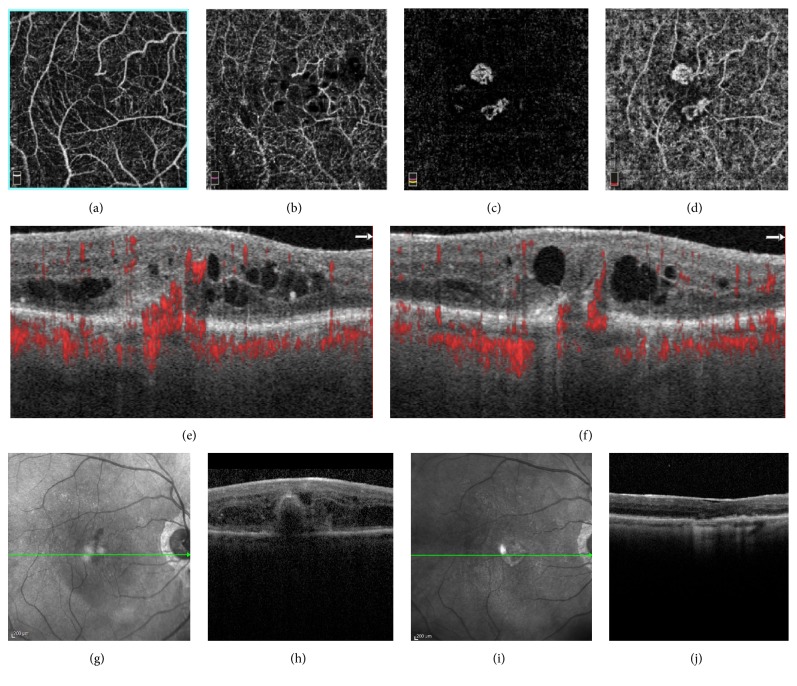
(a)-(d) Optical coherence tomography angiography images (6 × 6 mm) revealed the progression of the tuft-shaped lesion from the superficial layer to choroid capillary. (a) Superficial capillary plexus segmentation. (b) Deep capillary plexus segmentation. (c) Outer retinal layer segmentation. (d) Choriocapillaris segmentation. (e)-(f) B-Scans optical coherence tomography angiography showed clearly two distinct intraretinal neovascularizations originated in the superficial layer descending into a tuft-shaped lesion toward the sub-RPE space, respectively above (e) and under (f) the fovea. (g)–(h) Spectral Domain-Optical Coherence Tomography at baseline. (i)–(j) Spectral Domain-Optical Coherence Tomography after treatment.

Fundus examination revealed macular hemorrhage, exudates and intraretinal edema in the right eye (RE); these clinical findings supported the diagnosis of type 3 CNV in the RE that was then confirmed by SD-OCT and OCTA imaging. OCTA showed clearly two distinct intraretinal neovascularizations located in the segmentation of choroid capillary, both bordering the sub-RPE space (Figure 2). We administered intravitreal injections of bevacizumab in the fellow eye. The patient received 10 intravitreal injections in the LE and 5 in the RE, with an improvement of 10 LN in visual acuity in both eyes.

To date, we decided to stop the anti-VEGF therapy because SD-OCT did not show any CNV activity in both eyes.

## 3. Discussion

CNV rarely occurs in patients affected by RP [15]. Only few cases of CNV have been described in the literature [15–19]. To date, two case of RP with RAP have been reported [19, 20]. Nagao et al firstly described a case of RP associated with RAP. Recently Sayadi et al reported another similar case of type 3 neovascularization associated with RP.

The underlying pathogenesis of RP-associated CNV remains uncertain. Malik et al hypothesized that retinal pigment epithelium changes, constantly present in retinitis pigmentosa, associated with concomitant photoreceptor cell involvement and chorio-capillaris damage could lead to development of CNV [3]. We decided to perform genetic analysis to search for genetic variants correlated with the disease phenotype displayed by the patient.

Genetic testing revealed the presence of two variants (c.1265C>T and c.1162T>C) in* SEMA4A* and* CNGA1*, respectively. Both genes are known to be associated with autosomal recessive RP. However, the clinical significance of these variants is ambiguous, and cannot be utilized to provide a certain genetic diagnosis of RP in this case. Further studies will be probably decisive to clarify the genetic background of peculiar phenotypes and of RP in general.

Concerning the AMD screening, the patient revealed a moderate risk to develop the disease (heterozygous genotypes for* ARMS2* and* IL-8* susceptibility variants).* ARMS2* and* IL-8 *have also been associated with angiogenesis, Extra-Cellular Matrix (ECM) organization, alteration of Bruch's membrane, inflammatory and immune response overactivation observed in AMD [13, 14]. In addition, recent studies suggested that* SEMA4A* may play a critical role in immune response activation and angiogenesis [10]. Interestingly, previous studies correlated a decreased expression of* CNGA1 *and other phototransduction related-genes to more severe AMD, especially the neovascular form of disease [14, 21]. Whether the identified VUS will be classified as pathogenic or not,* SEMA4A* and* CNGA1* share some biological pathways with* ARMS2* and* IL-*8, suggesting that these genes may interact together and contribute to increase the susceptibility to develop neovascular lesions, which may be more facilitated to grow in RP cases because of photoreceptor cells degeneration, RPE changes and choriocapillaris damage.

Our study represents the first description of a bilateral type 3 neovascularization in a patient with RP genetically determined.

Because of the rarity of the condition, limited experience about the effective treatment for RP-related CNV has been accrued. Laser photocoagulation [15], photodynamic therapy [16], and intravitreal injections [3, 18] have been proposed as available therapies for CNVs complicating RP. Intravitreal bevacizumab has been reported to be effective for CNV stabilization in patients with RP. Malik et al reported a successful treatment of CNV in RP with a single intravitreal bevacizumab injection [3]. Nevertheless, Battaglia Parodi et al reported a longer period of treatment to achieve CNV stabilization in patients with RP [18]. Recently Sayadi et al reported a case of type 3 neovascularization associated with RP treated with 8 intravitreal injections of ranibizumab during the first year of follow-up, suggesting that anatomical stabilization of RP-related CNV may be difficult to reach.

Despite the rarity of condition, we reported to date the first case of development of a bilateral type 3 neovascularization associated with RP. Given the age of the patient, we cannot certainly exclude that the simultaneous presence of the two pathologies shown by the patient (RP and CNV) may be purely coincidental. Further research is required to examine the mechanism associating these two conditions and to establish the effective treatment for RP-related CNVs.
